# Doxorubicin-related effects on cardiorespiratory function and body composition

**DOI:** 10.1016/j.ahjo.2024.100360

**Published:** 2024-01-08

**Authors:** Ashley J. Smuder

**Affiliations:** Department of Applied Physiology and Kinesiology, College of Health and Human Performance, University of Florida, Gainesville, FL, USA

**Keywords:** Cardio-oncology, Exercise, Chemotherapy

Doxorubicin (DOX) is a highly effective chemotherapeutic agent used in the treatment of many solid tumor, blood and gynecological cancers. However, its use is associated with both acute and chronic adverse cardiovascular events which negatively impact cancer patient quality of life and long-term survivorship [1]. Although there are cardiovascular differences between rodents and humans, primarily related to the large variation in heart rate across species, there are distinct advantages to utilizing small rodents to model human disease [2]. In the case of DOX chemotoxicity, the ability to assess cardiac damage, cardiorespiratory capacity and body composition has allowed for improvements in the translational potential of rodent models of acute and chronic DOX toxicity, while also allowing for investigation at the molecular level [1]. In addition, the smaller amount of drug and shorter time period needed for these studies allows for increased productivity in understanding the mechanisms of DOX cardiotoxicity and in testing potential therapeutic countermeasures [3].

Utilizing a rat model where DOX was administered every three weeks for a total of four cycles (5.7 mg/kg/cycle intravenously), our team was able to test the effect of two exercise prescriptions on cardiorespiratory and body composition outcomes. This novel model was adapted based on clinical DOX prescription and addresses several limitations of DOX rodent dosing protocols including dose administered, frequency of administration and route of administration [1]. The dose was scaled based on established dose conversion calculations between rats and humans [4] and equates to 40 mg/m^2^ each cycle for a cumulative dose of 160 mg/m^2^. The exercise prescriptions utilized were based on current clinical trials utilizing cycling and treadmill exercise modalities designed to determine the efficacy of concomitant exercise to reduce adverse side effects of chemotherapy treatment in breast cancer patients [5] and included both a moderate intensity exercise training protocol (treadmill running, 1-h/day, 3 days/week, ~70 % VO_2_max) and a high-intensity interval training (HIIT) protocol (treadmill running, 4×4min intervals, 3 days/week, ~90 % VO_2_max). Initial results with this model indicate that exercise naïve rats mimic patient phenotype with relative fat mass significantly increasing over the course of treatment. In contrast, both exercise training prescriptions reduced relative fat mass and the HIIT rats showed an increase in relative lean mass. Exercise tolerance testing did not reveal a significant increase when comparing pre-treatment times to one-week post-treatment, but the moderate intensity group did have an absolute time change that was greater than the sedentary, DOX-treated rats.

These findings are similar to patient outcomes related to body composition and changes in exercise tolerance [6,7], and thus demonstrate the relevance of this novel rat model of DOX chemotherapy treatment. In addition, they show the feasibility and benefit of including exercise training as adjuvant therapy. Future application of this model is needed to continue verifying its translatability through the assessment of acute and chronic effects of DOX treatment and exercise interventions on cardiac function outcomes.
